# Productivity of Concentration-Dependent Conversion of Substitutional Nitrogen Atoms into Nitrogen-Vacancy Quantum Emitters in Synthetic-Diamond by Ultrashort Laser Pulses

**DOI:** 10.3390/mi14071397

**Published:** 2023-07-09

**Authors:** Sergey Kudryashov, Pavel Danilov, Evgeny Kuzmin, Nikita Smirnov, Alexey Gorevoy, Victor Vins, Daniil Pomazkin, Petr Paholchuk, Andrey Muratov, Alexey Kirichenko, Nikolay Rodionov, Evgeny Vasil’ev

**Affiliations:** 1 Lebedev Physical Institute, 119991 Moscow, Russia; danilovpa@lebedev.ru (P.D.); kuzmine@lebedev.ru (E.K.); smirnovna@lebedev.ru (N.S.); a.gorevoy@lebedev.ru (A.G.); vgvins@gmail.com (V.V.); d.pomazkin@lebedev.ru (D.P.); p.paholchuk@lebedev.ru (P.P.); muratovav@lebedev.ru (A.M.); 2 LLC VELMAN, 630058 Novosibirsk, Russia; 3 Institution “Project Center ITER”, 123182 Moscow, Russia; akir73@mail.ru (A.K.); n.rodionov@iterrf.ru (N.R.); 4 Saint-Petersburg Mining University, 199106 Saint-Petersburg, Russia; vasilev_ea@pers.spmi.ru

**Keywords:** HPHT diamond, substitutional nitrogen atoms, femtosecond laser, filaments, 3D-scanning Raman and photoluminescence micro-spectroscopy, optically active radiation defects, conversion to NV-centers

## Abstract

Tightly focused 515-nm, 0.3-ps laser pulses modify in a laser filamentation regime the crystalline structure of an Ib-type high-pressure, high-temperature (HPHT) synthesized diamond in a thin-plate form. The modified microregions (micromarks) in the yellow and colorless crystal zones, possessing different concentrations of elementary substitutional nitrogen (N) impurity atoms (C-centers), exhibit their strongly diminished local IR absorption (upon correction to the thickness scaling factor). Simultaneously, local visible-range (400–550 nm) absorption coefficients were increased, and photoluminescence (PL) yield was strongly enhanced in the broad range of 450–800 nm. The strong yellow-red PL enhancement saturates with laser exposure, implying the complete conversion of C-centers into nitrogen-vacancy (NV^0,−^) ones due to the laser-induced generation of Frenkel “interstitial-vacancy” I–V carbon pairs. The other emerging blue-green (>470 nm) and green-yellow (>500 nm) PL bands were also simultaneously saturated versus the laser exposure. The observed IR/optical absorption and PL spectral changes enlighten the ultrashort pulse laser inscription of NV^0−^-based quantum-emitter centers in synthetic diamonds and enable the evaluation of the productivity of their inscription along with the corresponding I–V generation rates.

## 1. Introduction

Since 2017, ultrashort-pulse laser inscription of photoluminescent NV-type color centers in high-purity synthetic diamonds is considered the most versatile technology for robust, controllable fabrication of NV^0^-based single-photon sources for quantum optics applications [1,2]. The underlying physical mechanism was supposed to be an attachment of laser-generated vacancy to the ultrarare (order of ppb) single-atom substitutional nitrogen impurities (color C-centers, N_S_) in electronic-grade diamonds [3]. This mechanism and its experimental implementation regimes were explored in a number of recent studies [4,5,6], considering the characteristic NV^0^-center spontaneous emission (photoluminescence, PL) at the 575 nm zero-phonon line (ZPL) along with its phonon progressions [7] as a function of laser pulse width and pulse energy [8,9], as well as exposure [10]. Meanwhile, the other important parameter is C-center concentration [C], which could strongly modify NV^0^-center emission in favor of its negatively charged form (NV^−^-center, ZPL at 637 nm [7]) due to the n-doping effect of C-centers in the diamond lattice. Moreover, the relative productivity of NV-center generation by ultrashort laser pulses in diamonds per one Joule of laser energy and one photo-generated electron-hole pair were not also studied yet in a straightforward manner. Some previous estimates—e.g., in single-NV-center generation experiments [3,4,5,6,8,10]—could work as the bottom limit for such productivity, requiring for certain [C] range to be utilized to demonstrate saturation of PL yield of NV-centers for the given initial [C] values. Moreover, optimal range of [C], enabling the maximal PL yield for NV^0^-centers without charging till NV^−^-centers or structural aggregation till H3-centers (2NV) [11], as well as the important laser-induced generation rate of carbon “interstitial-vacancy (I–V)” pairs in the host carbon lattice and their role in structural reconfiguration of color center are still under study [3,4,5,6,7,8,9,10,11,12,13], thus hindering potential important applications in diamond photonics.

In this study, we inscribed photoluminescent micromarks in the bulk of an Ib-type synthetic diamond with a spatially inhomogeneous known content of exceptionally nitrogen-impurity C-type centers, using ultrashort laser pulses of variable energy and exposure in the filamentation regime. The inscribed photoluminescent micromarks were characterized by differential Fourier-transform IR (FT-IR) and optical transmission microspectroscopy, 3D-scanning confocal Raman, and photoluminescence microspectroscopy to visualize the laser-induced N→NV transformations both qualitatively and quantitatively, in order to estimate the underlying I–V and NV-center productivity characteristics.

## 2. Materials and Methods

**Figure 1:**
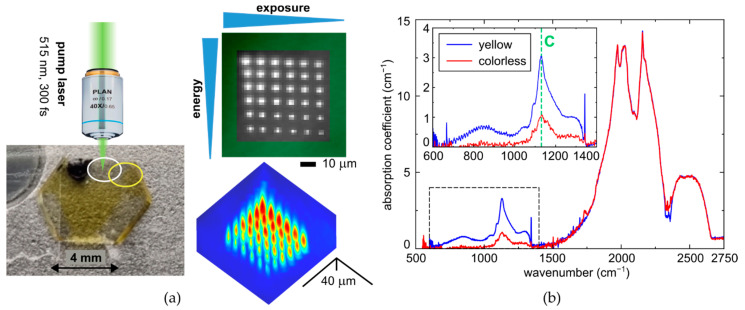
(**a**) Layout of laser inscription scheme with an optical image of the HPHT plate with its used color zones, differing in C-center content. Two-dimensional and three-dimensional PL microimages of micromark arrays inside the plate. (**b**) FT-IR spectra of the colorless and yellow zones in the plate, showing their depth-averaged C-center concentrations of 22 and 70 ppm. Spectral assignment after [7,15,16,17].

In this work, we use a workstation for 3D-micro and nanostructuring (Figure 1a) based on a femtosecond Yb-doped fiber laser system Satsuma (Amplitude Systemes, Pessac, France), operating at the fundamental wavelength λ = 1030 nm and second harmonic (SH) wavelength λ = 515 nm (TEM00-mode), pulse duration τ ≈ 0.3 ps, variable pulse energy E = 0.01–10 μJ and repetition rate f = 0–500 kHz. The SH laser pulses were focused by a 0.65-NA micro-objective lens into the spot size w_0_ = 0.6 ± 0.1μm (1/e-intensity radius) at the refraction-index (*n* ≈ 2.4) corrected depth of ∼ 100–250 µm inside a diamond plate. Due to the high refractive index value of the diamond, the focusing geometry is defined by a significantly lower effective NA = 0.65/2.4 ≈ 0.27 and a proportionally longer Rayleigh range. The sample was mounted onto a PC-driven three-dimensional motorized translation stage (Prior, London, UK) and exposed at the fixed depth in its bulk in a series of microregions as a function of the pulse energy E = 0.15–1 μJ and exposure time T ≈ 10–320 s at the 100-kHz repetition rate (pulse exposure N ≈ (1–32) × 10^6^ pulses). Taking into account the transmission of optical components (about 0.6 at 515 nm), the delivered pulse energy (0.09–0.6 μJ) was in most cases above the threshold energy for filamentation at the pulse width, 0.21 μJ [14] (see also the elongated micromarks in Figure 1a, typical energy-dependent lengths—15–40 μm, see also longitudinal PL profiles in Section 3.2). The peak laser power P = E/τ ≈ 0.3–2 MW was also well above the known magnitudes for critical power of self-focusing at the wavelength, 0.5 MW [14], while peak fluence and peak intensity evaluated for the linear focusing regime (effective for E < 0.21 μJ) would be ≈9–60 J/cm^2^, ≈ 30–200 TW/cm^2^, respectively. The high pulse energies were used in order to produce distinct PL micromarks over the reasonable exposure times of 10–320 s at the moderate pulse repetition rate of 100 kHz without considerable thermal lensing.

The experimental sample was an inhomogeneously colored synthetic (high-pressure, high-temperature, HPHT) Ib-type diamond hexagonal plate (hexagon side—4 mm, thickness D_0_ ≈ 0.5 mm) (Figure 1a), possessing exceptionally C-centers with their local concentrations [C_y_] ≈ 70 ppm or ≈1.2 × 10^19^ cm^−3^ (yellow region) and [C_c_] ≈ 22 ppm or ≈3.7 × 10^18^ cm^−3^ (colorless region), according to FT-IR microspectroscopic measurements, [C(ppm)] = 25 × α_1135_ (cm^−1^) [7] or [C(ppm)] ≈ (21–22) × α_1130_ (cm^−1^) at higher [C] [15] in range of 500–4000 cm^−1^ (Figure 1b), using a FT-IR spectrometer Optics IFS-125HR with a microscope Hyperion 2000 (Bruker, Billerica, MA, USA) and a FT-IR spectrometer Vertex V-70 with a microscope Hyperion 1000 (Bruker, Billerica, MA, USA).

**Figure 2:**
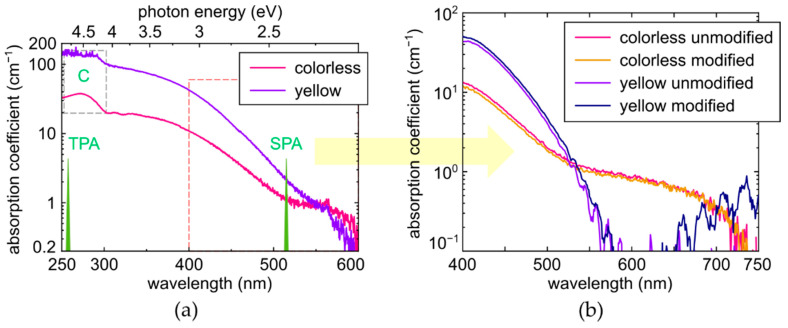
(**a**) Optical absorption coefficient spectra of the colorless (pink curve) and yellow (violet curve) zones inside the diamond plate, showing UV-absorption of C-centers and position of single (SPA)/two-photon (TPA) 515 nm laser absorption. Spectral assignment after [7,18,19]. (**b**) Optical absorption coefficient spectra of laser-modified spots relative to the reference unmodified ones in the colorless (pink and orange curves) and yellow (violet and blue curves) regions.

The HPHT plate was characterized by an ultraviolet(UV)-near-IR (190–1100 nm) transmission spectrophotometer SF-2000 (OKB Spektr, St. Petersburg, Russia) (Figure 2a) and an ultraviolet(UV)-near-IR (350–900 nm) transmission microscope-spectrometer MFUK (LOMO, St. Petersburg, Russia) (Figure 2b). For both the colorless and yellow zones, there is a sharp absorption edge near 600 nm, with the absorption coefficient gradually rising toward 270 nm (the absorption band of C-centers [7,18,19]). The band is clearly visible for the colorless region, but it is not fully distinguishable for the yellow region (see also Figure S1 in the Supplementary Materials file for linear scale plots). Indeed, according to the data provided in [19] and other approximate relationships for C-centers in [7] (α_270nm_ ≈ (21–45)α_1135cm_^−1^, α_477nm_ ≈ 1.4α_1135cm_^−1^), the absorption coefficient for [C_y_] ≈ 70 ppm would exceed 150 cm^−1^, which was beyond the sensitivity threshold of the spectrophotometer used. Due to the same reason, the absorption edge at around 225–235 nm (indirect bandgap absorption) for the yellow zone is not as pronounced as for the colorless one. Therefore, we did not use the UV transmission data in the 270 nm band for the quantitative analysis of the concentration of C-centers.

**Figure 3:**
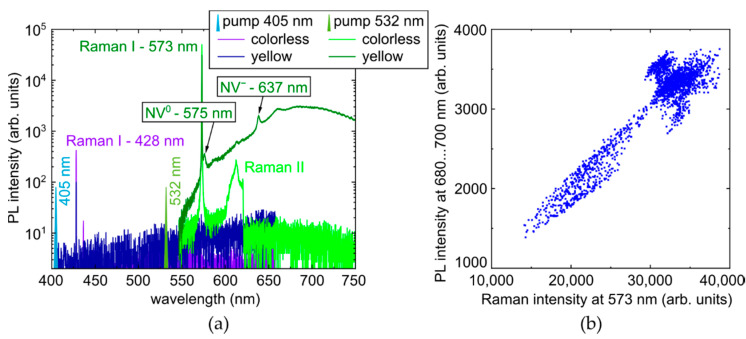
(**a**) PL spectra of the unmodified diamond at the 405 nm and 532 nm photoexcitation in the colorless and yellow regions (spectral assignment after [7,20,21]). (**b**) Correlation of 573 nm Raman and 680 nm PL peak intensities for the unmodified spot of the yellow region, used for Raman correction of PL spectra.

Additional complementary characterization was provided by room-temperature photoluminescence (RT PL) spectroscopy of the sample, using a 3D-scanning confocal Raman/PL microscope-spectrometer Confotec 350 (SOL instruments, Minsk, Belorussia) and inVia InSpect (Renishaw, London, UK) with the measured confocal distance about 30–40 μm. The colorless sample regions exhibit at the 405 nm and 532 nm continuous-wave (CW) laser excitation characteristic Raman lines of the carbon lattice at 428 nm and 573 nm (Figure 3a, also second-order Raman band at 532 nm excitation), respectively, considerably broadened due to the utilized moderate nominal resolution of 2.5 cm^−1^. The yellow regions demonstrate just the Raman line at 428 nm at the 405 nm excitation, but in contrast, characteristic PL bands of both nitrogen-related NV^0^-(zero-phonon line, ZPL—575 nm [7,20]) and NV^−^-(ZPL—637 nm [7,21]) centers. To account for sample absorption at the pump wavelengths and focusing conditions inside the sample, the peak intensities of the Raman lines were used for correcting corresponding PL intensities at the same pump wavelength for different probed depths inside the diamond. The validity of this correction is confirmed by the correlation between the Raman line intensity and the PL intensity in the 680–700 nm band for the unmodified part of the yellow zone (see Figure 3b). Moreover, the 3D-scanning mode was utilized to evaluate thickness values, D, of laser-modified regions in the diamond (see, e.g., Figure 1a), used, e.g., for deconvolution of their FT-IR absorption averaged over the whole sample thickness D_0_ ≈ 0.5 mm.

## 3. Experimental Results

### 3.1. Variation in Mid-IR and Optical Absorption of Diamond Color Centers in Micromarks

**Figure 4:**
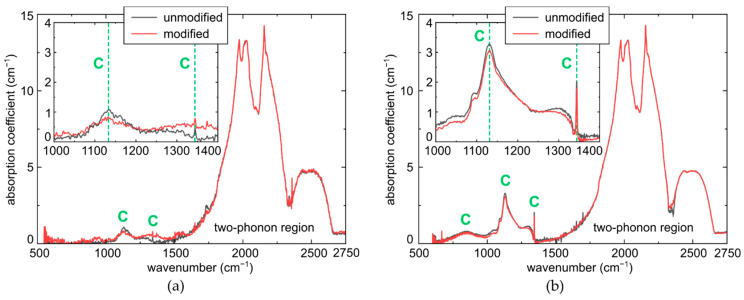
FT-IR spectra of laser-modified regions (inscription parameters E = 0.75 μJ, T = 20 s) relative to the reference unmodified ones in the colorless (**a**) and yellow (**b**) regions (spectral assignment of C-center absorption bands after [7,15,16,17]).

FT-IR microspectroscopy of the laser-modified square regions in the colorless and yellow diamond regions (Figure 4) indicates the considerable, almost complete local decrease of the concentrations [C] (main peak at 1130 cm^−1^ and the related peak at 1344 cm^−1^ [7,15,16,17]), comparing to the unmodified regions. This fact qualitatively demonstrates the complete structural transformation of these color centers upon laser exposure and, upon systematic studies, enables quantitative evaluation of the related interstitial vacancy and NV-center production. However, since such laser inscription of multiple energy- and exposure-dependent sub-mm-sized square regions of the laser-modified diamond is a time- and material-consuming procedure, our productivity analysis was performed below for PL intensities of NV-centers and other I, V-related spectral features.

Specifically, accounting for the micromark thickness magnitudes D ≈ 30 μm (see below in Section 3.2), one can evaluate the resulting C-center depletion in the micromarks as Δα(C)/D = α(C)/D_0_. In the yellow diamond region with lower noise, Δα(C) ≈ 0.2 cm^−1^ (Δ[C] ≈ 4 ppm, Figure 4b), corresponding to α(C) ≈ 3 cm^−1^ and Δ[C] ≤ 66 ppm, comparable to [C_y_] ≈ 70 ppm or ≈1.2 × 10^19^ cm^−3^, thus implying the almost complete laser-induced conversion of C-centers. In the colorless region with a much lower concentration [C_c_] ≈ 22 ppm or ≈3.7 × 10^18^ cm^−3^, one could assume an even more complete conversion.

Quite similar changes occurred in the spectra of the optical absorption coefficient (Figure 2), acquired by the optical transmission micro-spectroscopy in the range of 400–750 nm (Figure 2b). Here, the main change in the absorption occurs as the minor (sample depth-averaged, see the factor D_0_/D above) raise for the laser-modified material in the absorption band below 500 nm (initially—C-center absorption [7,18,19], see Figure 2a). Meanwhile, since we see the strong depletion of [C] in FT-IR spectra for the laser-modified regions (Figure 4), one could assign this minor raise of the absorption coefficient to laser generation of common radiation point defects, like pure vacancy V^0^ (410–430, 741 nm/GR1-GR8), N2V (N + 2V, ZPL at 440.3 nm), NnI (N + nI, 441.6 nm), 2I2V (2I + 2V, 469.9 nm/TR12), I2V (I + 2V, 503.5 nm/3H) and 2NV (2N + V, 503.2 nm, H3) centers [7,22,23,24,25,26,27], strongly quenched without annealing under our irradiation conditions. Then, despite the laser-induced diminishing of the blue-green C-center absorption, additional absorption in the spectral range in Figure 2b could come from the radiation defects. Specifically, in the colorless region, the absorption coefficient at 400 nm even decreases by 1 cm^−1^ (−8%), while in the yellow region, it increases at 400 nm by +5 cm^−1^ (+10%), with the relative changes compared to the factor D_0_/D ~ 10.

### 3.2. Power- and Exposure-Dependent Variation in PL Spectra of Diamond Color Centers in Micromarks

#### 3.2.1. General View of PL Spectra

**Figure 5:**
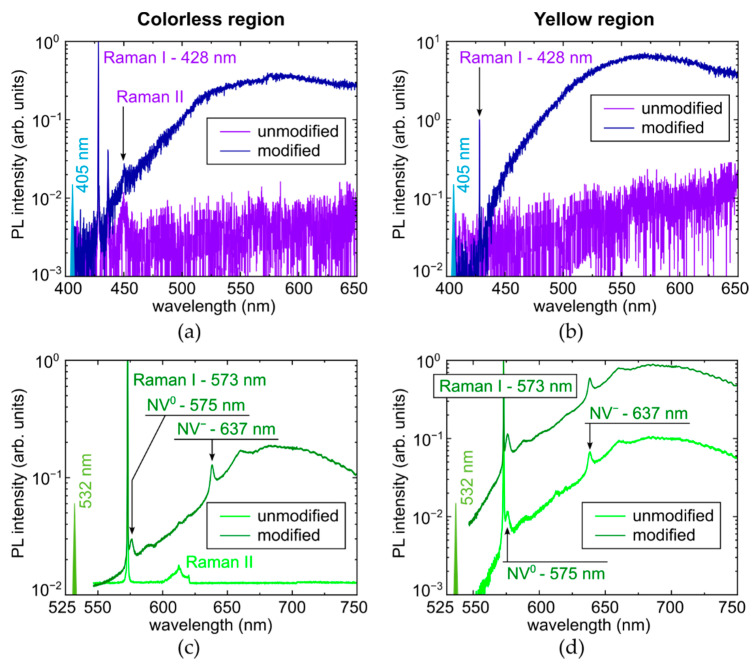
Room-temperature PL spectra of micromark in colorless (left column—(**a**,**c**)) and yellow (right column—(**b**,**d**)) regions, excited at 405 nm (top row—(**a**,**b**)) and 532 nm (bottom row—(**c**,**d**)) wavelength. Spectral assignment of Raman I, II, and NV lines after [7,20,21].

Raman-corrected PL studies provide the facile acquisition of the laser-induced micro-modification in the diamond as a function of laser energy and exposure (see figures in Section 3.2.2, Section 3.2.3 and Section 3.2.4 below). Here, we will consider the qualitative view of the representative Raman-corrected PL spectra excited at the 405 nm and 532 nm wavelengths in the lower-nitrogen colorless (Figure 5a,c and Figure S2a,c) and higher-nitrogen yellow regions (Figure 5b,d and Figure S2b,d). Importantly, at both these pump laser wavelengths, the Raman I peak, representing Stokes Raman first-order scattering via the optical phonon, remains almost unchanged for the laser-modified and unmodified regions in the samples, i.e., the carbon lattice order remains the same upon the laser modification.

Specifically, one can observe, in addition to the first-order and second-order Raman lines at 428 nm (full-width at a half maximum, FWHM ≈ 10 cm^−1^ in the colorless region and ≈9 cm^−1^ in the yellow region) and about 450 nm, respectively, just noise in the background PL intensity of the unmodified diamond regions excited at the 405 nm wavelength (Figure 5a,b). However, upon the laser modification, the emerged structureless PL starts in the spectra from 425 nm and extends behind 650 nm, rapidly increasing till ≈ 500 nm and then changing rather slowly, without distinct ZPL features, with 428 nm Raman FWHM ≈ 12 cm^−1^ in both the colorless and yellow regions. The resulting peak PL intensities are ≈16-fold higher in the yellow region. The induced PL band could be potentially related to overlapping, unresolved separate bands of radiation-induced centers—pure vacancy V^0^ (410–430, ZPL at 741 nm/GR1-GR8), N2V (ZPL at 440.3 nm), NnI (ZPL at 441.6 nm), 2I2V (ZPL at 469.9 nm/TR12), I2V (ZPL at 503.5 nm/3H), 2NV (ZPL at 503.2 nm, H3), NV^0^ (ZPL at 575 nm) and NV^−^ (ZPL at 637 nm) [7,20,21,22,23,24,25,26,27]. Unfortunately, the utilized fs-laser generation of defects during micromarking makes the novel color centers quenched (non-equilibrium), thus ruling out the opportunity for their qualitative assignment by means of the characteristic ZPL (more about the fs-laser modified lattice effects in diamonds see soon in [27]).

In contrast, at the 532 nm excitation, the background PL spectra exhibit just the first-order and second-order Raman lines at 573 nm (FWHM ≈ 10 cm^−1^ in the colorless region and ≈9 cm^−1^ in the yellow region) and about 610 nm, respectively, in the colorless region (Figure 5c), while in the yellow region both NV^0^- and NV^−^-centers are initially present with their ZPLs at 575 (FWHM ≈ 56 cm^−1^) and 637 nm (FWHM ≈ 67 cm^−1^) and related phonon progressions, in addition to the first-order Raman line (Figure 5d). Furthermore, upon the laser modification, the PL intensities of NV^0^- and NV^−^-centers are dramatically increasing in comparison to the background and Raman intensities (≈10-fold PL increase at 575 nm and 637 nm for the yellow region, anomalous FWHM ≈ 96 cm^−1^ and ≈ 72 cm^−1^, respectively, FWHM ≈ 70 cm^−1^ at 637 nm in the colorless region), resulting in the ratio ≈ 5:1 of their intensities for the yellow and colorless diamond regions. Below, the laser-induced PL intensity in the different spectral regions—450–500 nm (tentatively, related to GR2-GR8, NnI, N2V, TR12-centers), 500–575 nm (tentatively, related to 3H and H3-centers), and 575–750 nm (NV^0^- and NV^−^-centers)—will be analyzed quantitatively as a function of laser pulse energy and exposure.

**Figure 6:**
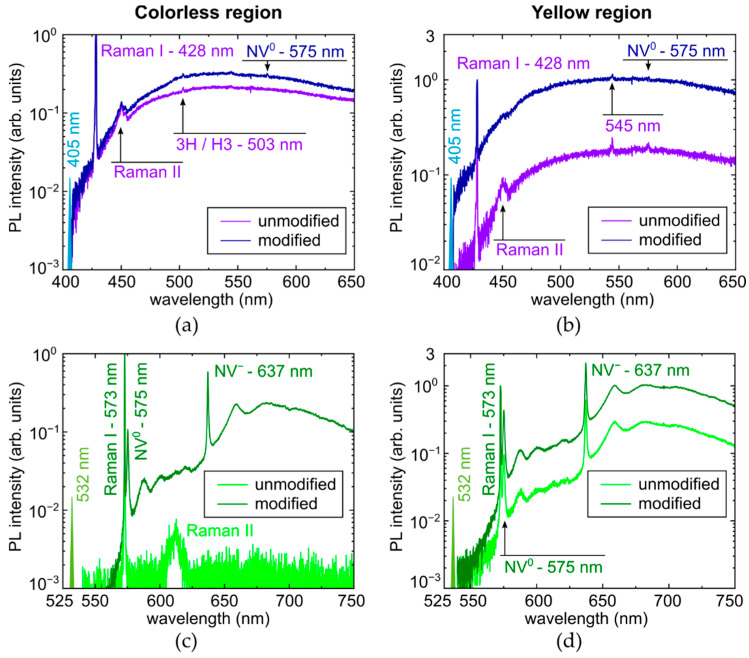
Liquid-nitrogen temperature (−190 °C) PL spectra of micromark in colorless (left column—(**a**,**c**)) and yellow (right column—(**b**,**d**)) regions, excited at 405 nm (top row—(**a**,**b**)) and 532 nm (bottom row—(**c**,**d**)) wavelength. Spectral assignment of Raman I, II, and NV lines after [7,20,21].

Surprisingly, low-temperature (−190 °C, liquid nitrogen cooling) PL studies demonstrated, in general, the same spectral features with some minor peaks of additional non-nitrogen impurities (Figure 6 and Figure S3). In particular, at 405 nm photoexcitation Raman FWHM ≈ 47 cm^−1^ in the colorless region and ≈32 cm^−1^ in the yellow region of the unmodified spots, while FWHM ≈ 50 cm^−1^ in the colorless region and ≈44 cm^−1^ in the yellow region of the laser-modified spots. Similarly, at 532 nm photoexcitation Raman FWHM ≈ 9 cm^−1^ in the colorless region and ≈17 cm^−1^ in the yellow region of both the unmodified and laser-modified spots. At this laser wavelength, FWHM ≈ 30 cm^−1^ and ≈18 cm^−1^ for NV^0^- and NV^−^-centers in the colorless region in the laser-modified spots, respectively, and in the yellow region for NV^0^- and NV^−^-centers FWHM ≈ 42 cm^−1^ and ≈25 cm^−1^ in the unmodified spots, while FWHM ≈ 36 cm^−1^ and ≈24 cm^−1^ in the laser-modified spots. The large measured FWHM parameters could have resulted from some icing during the measurements.

#### 3.2.2. Yellow-Red (>575 nm) PL Spectra Corrected to Raman Signal

**Figure 7:**
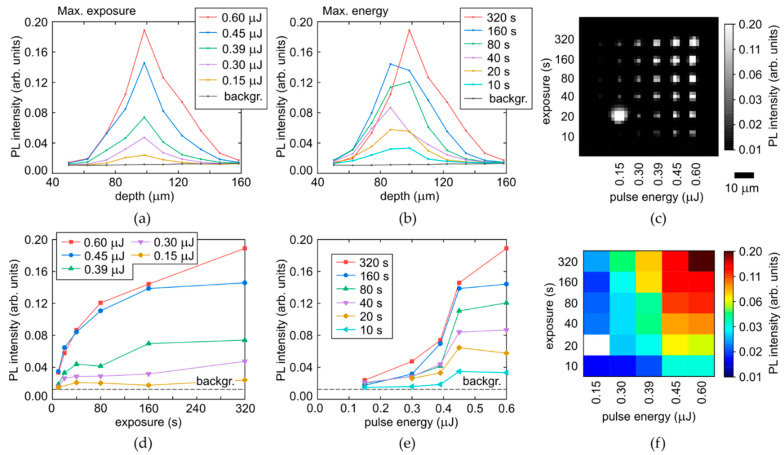
(**a**,**b**) Depth profiles of PL intensity at the selected wavelength of 680 nm in the colorless region of the diamond as a function of pulse energy and exposure, respectively, compared to background (backgr.). (**c**) 2D image (at the fixed depth) of micromark array at the 680 nm wavelength (the bright spot in the left down corner corresponds to the occasional breakdown facilitated by local mechanical defects or inclusions, once the laser energy and exposure were almost minimal in the series on this spot). (**d**,**e**) Dependences of maximal PL intensity (680 nm) on exposure and pulse energy, respectively. (**f**) 2D color map of maximal PL intensity (680 nm) vs. pulse energy and exposure.

**Figure 8:**
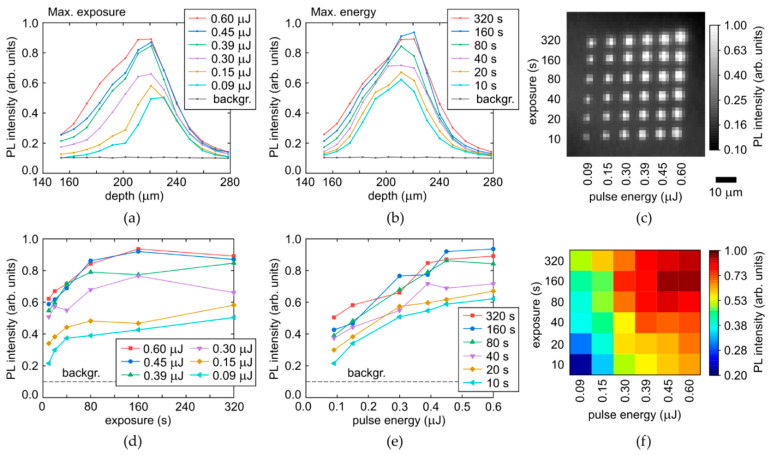
(**a**,**b**) Depth profiles of PL intensity at the selected wavelength of 680 nm in the yellow region of the diamond as a function of pulse energy and exposure, respectively, compared to background (backgr.). (**c**) 2D image (at the fixed depth) of micromark array at the 680 nm wavelength. (**d**,**e**) Dependences of maximal PL intensity (680 nm) on exposure and pulse energy, respectively. (**f**) 2D color map of maximal PL intensity (680 nm) vs. pulse energy and exposure.

The acquired spectra of the yellow-red PL vary versus laser pulse energy E and time exposure T only in their intensity, enabling to make spatial analysis for their most intense parts (680–700 nm, Figure 5c,d) upon the Raman correction in their arrays. The arrays are mapped at a moderate spatial resolution, resulting in pixelated images (Figure 7c and Figure 8c) and slightly noisy longitudinal profiles and parametric dependencies.

Specifically, the longitudinal profiles of micromarks in the array, shown for the refractive-index corrected depth (Figure 7 and Figure 8, top row), indicate the higher non-linearity of the PL intensity on laser pulse energy for the colorless region (the sharper profiles near the peak for the different energies, Figure 7a), comparing to the yellow region with its much smoother profiles (Figure 8a). This is well-consistent with the highly nonlinear PL rise versus E in Figure 7e, compared to the rather linear dependence in Figure 8e. Meanwhile, both these regions demonstrate the fast saturation of the maximum PL intensity in the micromarks versus T (Figure 7d and Figure 8d) with characteristic times T_sat,c_ = 40–80 s and T_sat,y_ = 20–40 s for the colorless and yellow regions, respectively, indicating complete local conversion of C-centers. The PL intensity growth rate for the yellow region is higher, being associated with the higher conversion rate and the 3-fold higher initial concentration of C-centers. The increase of the plateau level versus E in Figure 7d and Figure 8d reflects the spatial extension of the modified regions in the diamond (see Figure 1a, Figure 7a–c and Figure 8a–c). This level can also be saturated, when the volume with the maximum reachable NV-center density becomes larger than the confocal volume of the used 3D-scanning confocal microscope-spectrometers (one pixel in Figure 7c and Figure 8c).

#### 3.2.3. Blue-Green (450–500 nm) PL Spectra Corrected to Raman Signal

**Figure 9:**
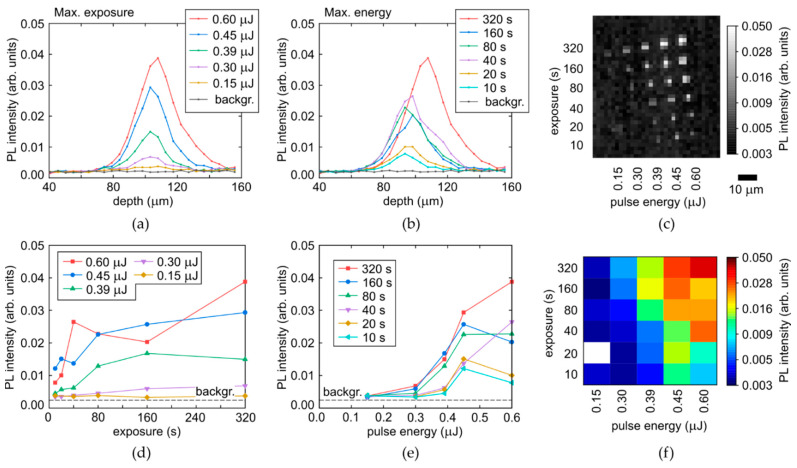
(**a**,**b**) Depth profiles of PL intensity at the selected wavelength of 475 nm in the colorless region of the diamond as a function of pulse energy and exposure, respectively, compared to background (backgr). (**c**) 2D image (at the fixed depth) of micromark array at the 475 nm wavelength. (**d**,**e**) Dependences of maximal PL intensity (475 nm) on exposure and pulse energy, respectively. (**f**) 2D color map of maximal PL intensity (475 nm) vs. pulse energy and exposure.

**Figure 10:**
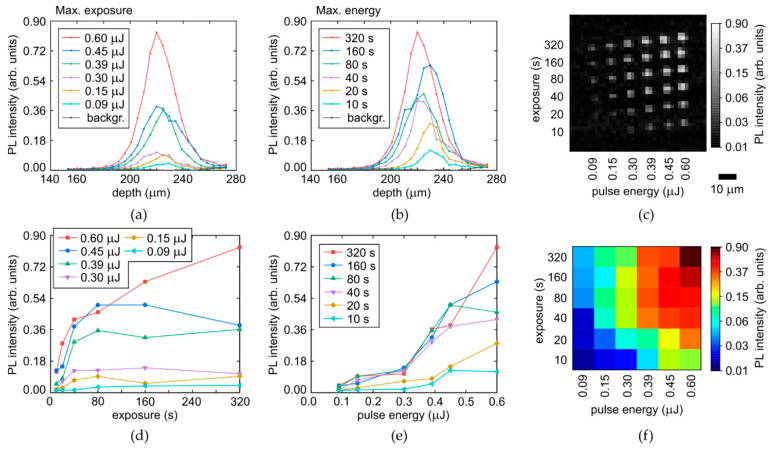
(**a**,**b**) Depth profiles of PL intensity at the selected wavelength of 475 nm in the yellow region of the diamond as a function of pulse energy and exposure, respectively, compared to background (backgr.). (**c**) 2D image (at the fixed depth) of micromark array at the 475 nm wavelength. (**d**,**e**) Dependences of maximal PL intensity (475 nm) on exposure and pulse energy, respectively. (**f**) 2D color map of maximal PL intensity (475 nm) vs. pulse energy and exposure.

Very similar trends were observed in both the diamond regions for the blue-green PL intensity in the range of 450–500 nm (Figure 9 and Figure 10), tentatively ascribed to radiation-induced centers—pure vacancy V^0^ (410–430 nm/GR2-GR8), NnI (ZPL—441.6 nm), N2V (ZPL—440.3 nm) and 2I2V (ZPL—469.9 nm/TR12) [7,22,23,24,25,26]. Specifically, one can find the same saturable PL intensity dependences on laser exposure with T_sat,c,y_ = 20–80 s (Figure 9d and Figure 10d), indicating the strong relationship of the blue-green PL emission and the exhausting of [C] in the laser-modified regions.

#### 3.2.4. Green-Yellow (500–575 nm) PL Spectra Corrected to Raman Signal

**Figure 11:**
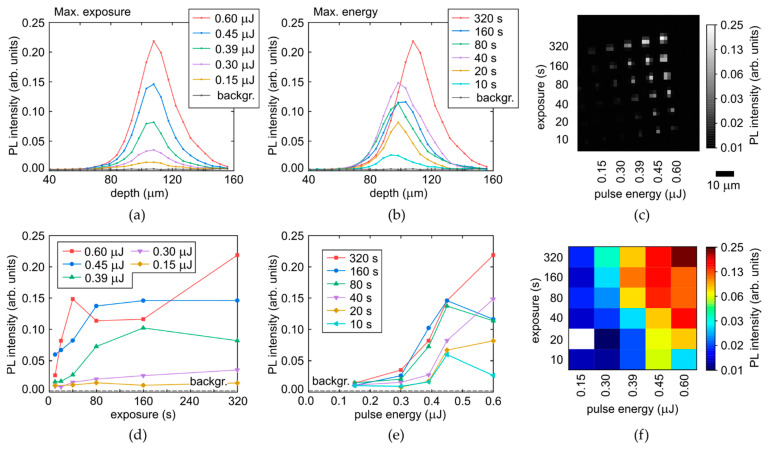
(**a**,**b**) Depth profiles of PL intensity at the selected wavelength of 525 nm in the colorless region of the diamond as a function of pulse energy and exposure, respectively, compared to background (backgr.). (**c**) 2D image (at the fixed depth) of micromark array at the 525 nm wavelength. (**d**,**e**) Dependences of maximal PL intensity (525 nm) on exposure and pulse energy, respectively. (**f**) 2D color map of maximal PL intensity (525 nm) vs. pulse energy and exposure.

**Figure 12:**
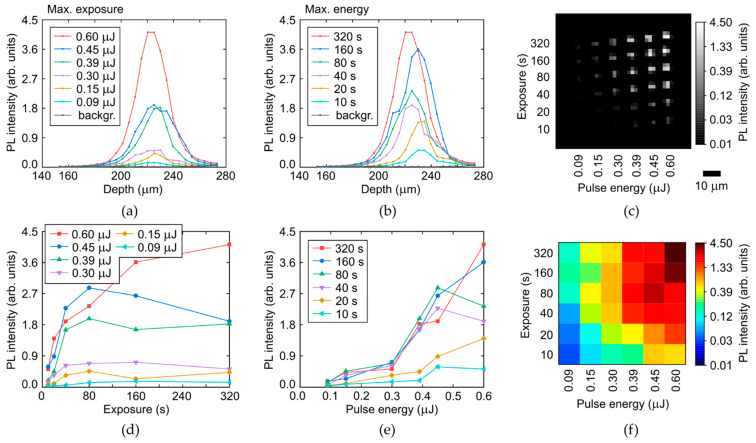
(**a**,**b**) Depth profiles of PL intensity at the selected wavelength of 525 nm in the yellow region of the diamond as a function of pulse energy and exposure, respectively, compared to background (backgr.). (**c**) 2D image (at the fixed depth) of micromark array at the 525 nm wavelength. (**d**,**e**) Dependences of maximal PL intensity (525 nm) on exposure and pulse energy, respectively. (**f**) 2D color map of maximal PL intensity (525 nm) vs. pulse energy and exposure.

Finally, very similar results were obtained in both the colorless and yellow regions of the diamond for the green-yellow (500–575 nm) PL intensity (Figure 11 and Figure 12), tentatively ascribed to radiation-induced 3H (ZPL—503.5 nm [7,24]) and H3 (ZPL—503.2 nm [7]) centers. Specifically, one can find the same saturation effect for the PL intensity dependences on laser exposure with T_sat,c,y_ *=* 20–80 s (Figure 11d and Figure 12d), indicating the strong relationship of the green-yellow PL emission and the exhausting of [C] in the laser-modified regions.

## 4. Discussion and Conclusions

In this study, we intentionally used the model Ib-diamond sample with its exclusive primitive atomic nitrogen C-centers in different concentrations and absent background visible-range photoluminescence (particularly, the PL-free colorless regions). This enabled us to explore the laser-induced C-center conversion and reveal in the PL spectra smooth unresolvable ultrabroad (470–800 nm) bands, making a few interesting findings:

(1) C-centers are quite significantly exhausted in the micromarks, as shown by the considerable change Δα(C) ~ 0.2 cm^−1^ in their IR absorption in the 1130-cm^−1^ peak (also, in the 1344-cm^−1^ peak, Figure 4), accounting for the thickness scaling factor D_0_/D ~ 10–20 for the sample thickness D_0_ ≈ 0.5 mm and typical micromark lengths D ~ 20–40 μm;
(2) Laser irradiation resulted in the rise of optical absorption in the range of 400–550 nm by 6–10% (Figure 2b), which should be multiplied by D_0_/D ~ 10–20 to evaluate true local changes. Such changes could be potentially related to different radiation-induced centers like pure vacancy V^0^ (410–430, 741 nm/GR1-GR8), N2V (ZPL at 440.3 nm), NnI (441.6 nm), 2I2V (469.9 nm/TR12 ), I2V (503.5 nm/3H) and 2NV (503.2 nm, H3), NV^0^ (575 nm) and NV^−^ (637 nm) [7,20,21,22,23,24,25,26,27] with their UV-red absorption [7,18,19], potentially emerging in the broadband PL spectra (Figure 5 and Figure 6). Since the initial [C_y_] ≈ 1.2 × 10^19^ cm^−3^ and [C_c_] ≈ 3.7 × 10^18^ cm^−3^, the concentration of I–V pairs for their conversion should be high too, providing these different anticipated aggregated forms of N, I, and V species;
(3) PL intensities over the entire acquired range of 400–750 nm increase with the increasing initial [C] in the colorless and yellow regions, and exhibit saturation versus laser exposure, indicating the exhausting of C-centers. At first glance, this could imply that the underlying laser-generated color centers are nitrogen-containing, but another point is that electron-hole plasma in Ib-diamond occurs via two-photon ionization of C-centers [27] in the intrinsic absorption band ≈ 260 nm [7,18,19];
(4) Considering that NV-centers make a considerable fraction of the different (e.g., possible N2V, NnI, 2NV, etc.) anticipated nitrogen-based centers, their production rate per laser pulse could be evaluated as productivity η_NV_ ~ {[C_c_],[C_y_]}/(f × T_sat,c,y_) ~ 10^12^–10^13^ NV/cm^3^ in the fs-laser filamentation regime at the repetition rate f = 100 kHz and T_sat_ = 20–80 s. This is consistent with the fs-laser fabrication of single-photon sources in high-purity IIa-diamond, based on the inscription of single NV-centers per micrometer-sized focal volumes [3,4,5];
(5) Photogeneration rate of I–V pairs, productivity η_IV_ ~ 10^13^–10^14^ pairs/cm^3^, in the fs-laser filamentation regime could be evaluated for the first time, being comparable, though somewhat higher, than the productivity η_NV_, accounting for the possible abovementioned multitude of the possible different resulting nitrogen-based and nitrogen-free centers;
(6) Once the filamentation of the tightly focused fs-laser pulses requires near-critical electron-hole plasma (ρ_eh_ ~ 10^21^ cm^−3^) to counterbalance strong Kerr self-focusing [28], the yield of I–V pairs per electron-hole pair evaluated for the first time appears to be as low as η_IV_/ρ_eh_ ~ 10^−7^–10^−8^. Such low yield could indicate the high degeneracy in the near-critical electron-hole plasma, correlating with its ultrahigh Fermi-like expansion speeds [11], and its interaction with the entire carbon lattice [27], while very marginal non-correlated hot carriers could locally disturb elementary cells, producing I–V pairs.

To conclude, our fs-laser inscription studies in the model Ib-diamond sample with the known concentrations of its primitive atomic nitrogen C-centers were devoted to relating their laser-driven consumption quantitatively to conversion into NV centers, requiring the vacancy attachment. It was revealed, for the first time, directly via IR and optical measurements of C-center absorption, and indirectly via NV-center PL acquisition, that very considerable (almost complete?) local exhausting of C-centers was linked to the NV-center formation, saturated versus laser exposure. Then, by dividing the initial C-center concentration by the number of laser pulses and near-critical plasma density, photogeneration rates of NV-centers and I–V pairs (productivities η_NV_, η_IV_), as well as the yield of I–V pairs per electron-hole pair were estimated by order of magnitude. These findings could enlighten the laser-induced formation of individual single-photon NV-based quantum emitters [29] and potential damage processes in bulk diamonds, including their graphitization [30].

## Data Availability

Additional data could be provided by the authors upon a special request.

## Supplementary Materials

The following supporting information can be downloaded at: https://www.mdpi.com/article/10.3390/mi14071397/s1. Figure S1. (a) Optical absorption coefficient spectra of the colorless (pink curve) and yellow (violet curve) zones inside the diamond plate, showing absorption of C-centers in 270-nm band and position of single (SPA)/two-photon (TPA) 515-nm laser absorption. (b) Optical absorption coefficient spectra of laser-modified spots relative to the reference unmodified ones in the colorless (pink and orange curves) and yellow (violet and blue curves) regions. Figure S2. Room-temperature photoluminescence spectra of micromark in colorless (left column—(a,c)) and yellow (right column—(b,d)) regions, excited at 405-nm (top row—(a,b)) and 532-nm (bottom row—(c,d)) wavelength. Figure S3. Liquid-nitrogen temperature (−190 °C) photoluminescence spectra of micromark in colorless (left column—(a,c)) and yellow (right column—(b,d)) regions, excited at 405-nm (top row—(a,b)) and 532-nm (bottom row—(c,d)) wavelength. Figure S4. Raman I peak in room-temperature photoluminescence spectra of micromark in colorless (a,c) and yellow (b,d) regions, excited at 405-nm (a,b) and 532-nm (c,d) wavelength. Table S1. Main characteristics of Raman I peak in room-temperature photoluminescence spectra.
