# Supplementary material for: Productivity of Concentration-Dependent Conversion of Substitutional Nitrogen Atoms into Nitrogen-Vacancy Quantum Emitters in Synthetic-Diamond by Ultrashort Laser Pulses
Source: Micromachines (Basel). 2023 Jul 9;14(7):1397. doi: 10.3390/mi14071397 (PMC10385978; doi:10.3390/mi14071397)
Supplement: Supplementary file 1 [file micromachines-14-01397-s001.zip › micromachines-2437921-supplementary.pdf]

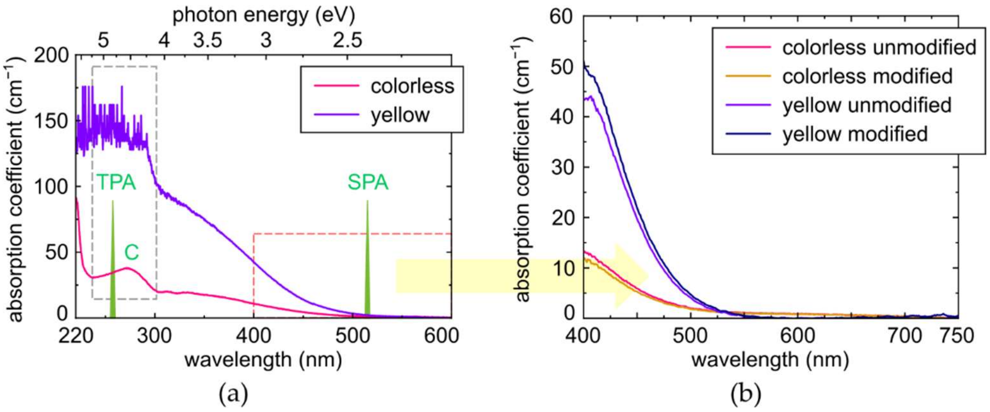

Figure S1. (a) Optical absorption coefficient spectra of the colorless (pink curve) and yellow (violet curve) zones inside the diamond plate, showing absorption of C-centers in 270-nm band and position of single (SPA)/two-photon (TPA) 515-nm laser absorption. **(b)** Optical absorption coefficient spectra of laser-modified spots relative to the reference unmodified ones in the colorless (pink and orange curves) and yellow (violet and blue curves) regions.

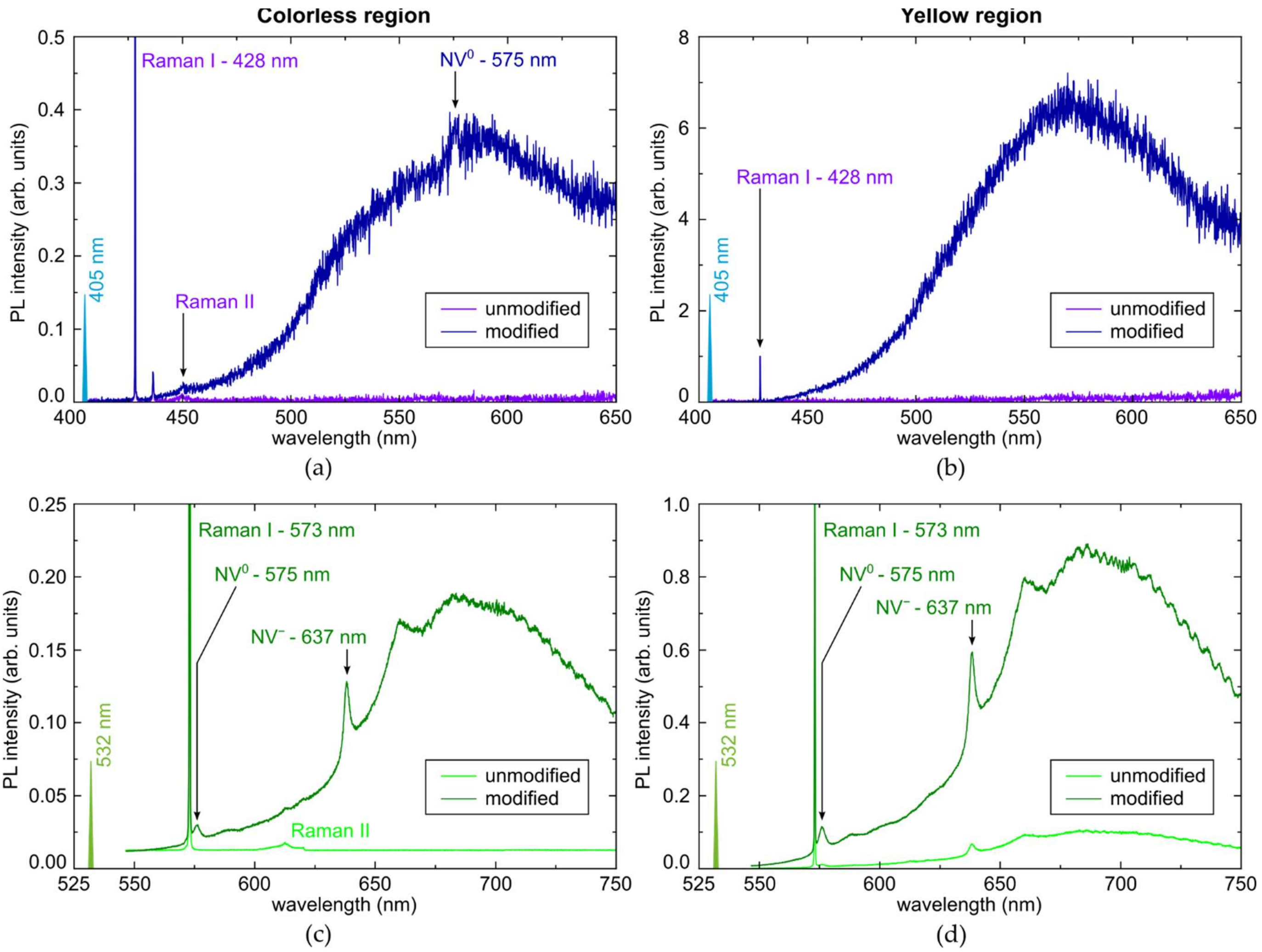

Figure S2. Room-temperature photoluminescence spectra of micromark in colorless (left column—(**a**,**c**)) and yellow (right column—(**b**,**d**)) regions, excited at 405-nm (top row—(**a**,**b**)) and 532-nm (bottom row—(**c**,**d**)) wavelength.

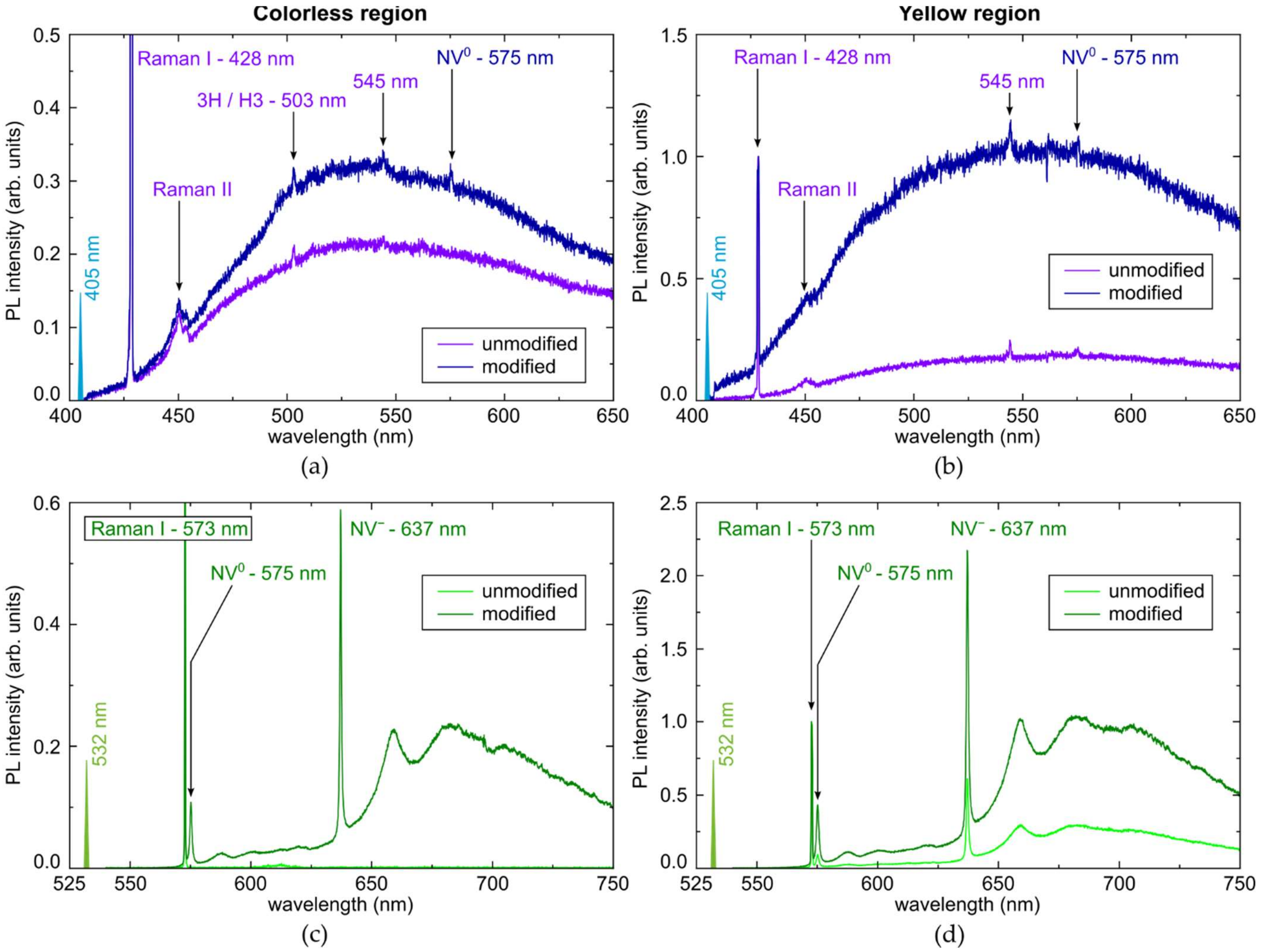

Figure S3. Liquid-nitrogen temperature (-190 °C) photoluminescence spectra of micromark in colorless (left column—(**a**,**c**)) and yellow (right column—(**b**,**d**)) regions, excited at 405-nm (top row—(**a**,**b**)) and 532-nm (bottom row—(**c**,**d**)) wavelength.

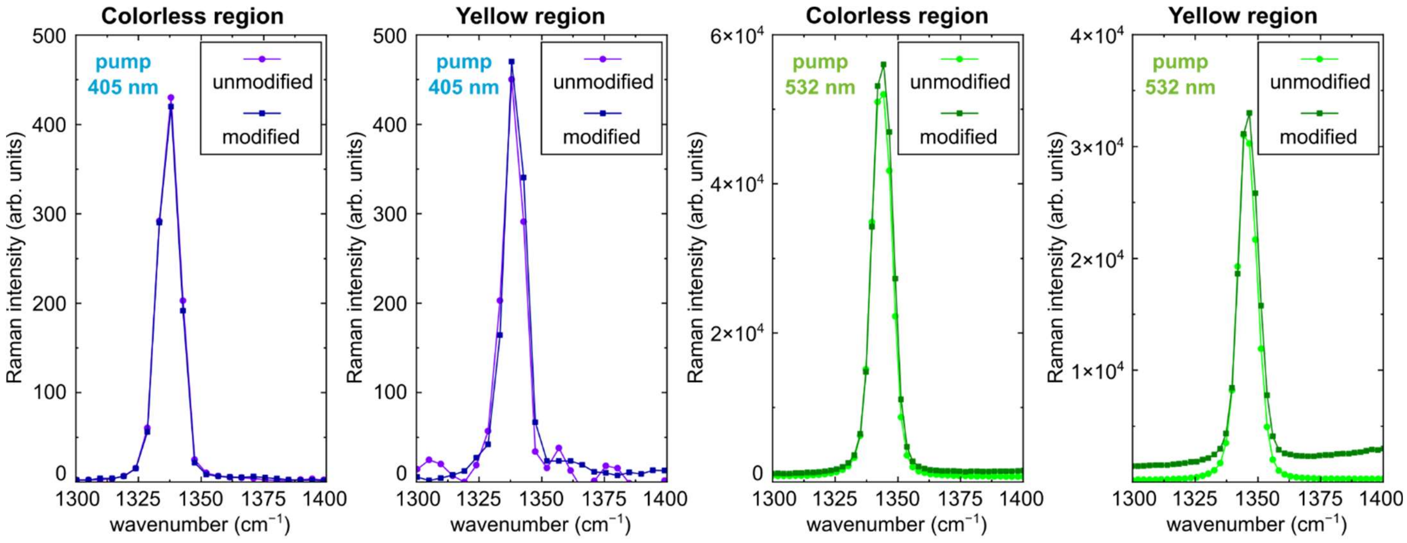

(a) (b) (c) (d)

Figure S4. Raman I peak in room-temperature photoluminescence spectra of micromark in colorless (**a**,**c**) and yellow (**b**,**d**) regions, excited at 405-nm (**a**,**b**) and 532-nm (**c**,**d**) wavelength.

Table S1. Main characteristics of Raman I peak in room-temperature photoluminescence spectra.

Region	Excitation wavelength, nm	Unmodified/ modified	Step*, cm^{-1}	Position, cm^{-1}	FWHM, cm^{-1}
colorless	405	unmodified	4.7	1337.2	10.2
		modified	4.7	1337.2	11.5
	532	unmodified	2.4	1343.5	9.6
		modified	2.4	1343.8	9.6
yellow	405	unmodified	4.7	1340.0	8.7
		modified	4.7	1340.0	11.7
	532	unmodified	2.4	1345.0	9.1
		modified	2.4	1345.0	9.5

* Spectrometer sampling step in the vicinity of the Raman peak.
